# Compilation of Secondary Metabolites from *Bidens pilosa* L

**DOI:** 10.3390/molecules16021070

**Published:** 2011-01-26

**Authors:** Fabiana Lima Silva, Dominique Corinne Hermine Fischer, Josean Fechine Tavares, Marcelo Sobral Silva, Petronio Filgueiras de Athayde-Filho, Jose Maria Barbosa-Filho

**Affiliations:** 1 Laboratório de Tecnologia Farmacêutica, Universidade Federal da Paraíba, Cx. Postal 5009, 58051-970, João Pessoa, PB, Brazil; E-Mails: josean@ltf.ufpb.br (J.F.T.); marcelo.ufpb@gmail.com (M.S.S.); athayde-filho@quimica.ufpb.br (P.F.A.F.); 2 Departamento de Farmácia, Faculdade de Ciências Farmacêuticas, Universidade de São Paulo, Av. Prof. Lineu Prestes, Bloco 15, 05580-900, São Paulo, SP, Brazil; E-Mail: domi@usp.br (D.C.H.F.)

**Keywords:** *Bidens pilosa*, Asteraceae, natural products, flavonoids, polyacetylenes

## Abstract

*Bidens pilosa* L. is a cosmopolitan annual herb, known for its traditional use in treating various diseases and thus much studied for the biological activity of its extracts, fractions and isolated compounds. Polyacetylenes and flavonoids, typical metabolite classes in the *Bidens* genus, predominate in the phytochemistry of *B. pilosa*. These classes of compounds have great taxonomic significance. In the Asteraceae family, the acetylene moiety is widely distributed in the Heliantheae tribe and some representatives, such as 1-phenylhepta-1,3,5-triyne, are noted for their biological activity and strong long-wave UV radiation absorbance. The flavonoids, specifically aurones and chalcones, have been reported as good sub-tribal level markers. Natural products from several other classes have also been isolated from different parts of *B. pilosa*. This review summarizes the available information on the 198 natural products isolated to date from *B. pilosa*.

## Introduction

The genus *Bidens* (Asteraceae: Heliantheae) comprises about 240 species with cosmopolitan distribution [1]. Many of these species have been investigated chemically to contribute to the classification of Asteraceae [2,3,4]. Interesting relationships within the Heliantheae, as well as its relationship with other tribes have been proposed on the basis of various types of compounds found in the tribe, especially acetylenes, sesquiterpene lactones and flavonoids [4,5]. The interest in these classes of compounds also has gone beyond chemotaxonomy. The biological activities, including antiparasitic, antifungal and antioxidant properties, of the predominant components in the tribe Heliantheae have been widely reported, and the investigation of these species for the discovery of new active compounds has expanded [6,7,8,9,10,11,12].

**Figure 1:**
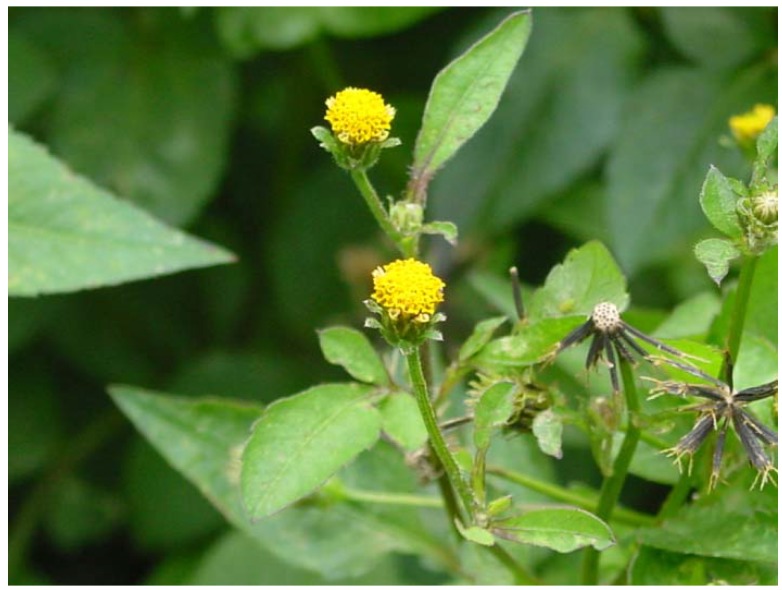
*Bidens pilosa* L.

*Bidens pilosa* L. (Figure 1) stands out among the species of the genus due to the large number of natural products characterized in it and the biological activities reported for its extracts, fractions and compounds. Therefore, in continuation of our research on bioactive molecules from the various species of the different families cited [13,14,15,16,17,18,19,20,21,22,23,24,25,26,27,28,29,30,31,32,33,34,35,36,37,38,39,40,41,42,43], we offer this compilation of the chemical constituents of *B. pilosa*. 

### *Bidens pilosa* L.

*B. pilosa* is an annual, erect and ruderal herb originating from South America and now found in almost all tropical and subtropical region countries [44,45,46]. It grows to a height of up to 1.5 m, branching from the base and its yellow flowers have 5-15 mm diameter [44,46]. 

It is a cosmopolitan herb, considered invasive of annual and perennial crops and widely distributed in disturbed areas and along roadsides in tropical and subtropical climates [46]. Nevertheless, this plant is commonly used in the traditional medicine. In Martinique, the decoction of the whole plant is used for its anti-inflammatory and hypoglycemic effects [47]. Aqueous preparations of the leaves are used by Zulu people for the treatment of dysentery, diarrhea and colic [48]. *B. pilosa* has been popularly used in China as a herbal tea ingredient or in traditional medicine for treating various disorders, such as diabetes, inflammation, enteritis, bacillary dysentery and pharyngitis [49]. In Brazil, it is widely used as a folk medicine by indigenous people to treat a variety of illnesses including pain, fever, angina, diabetes, edema, infections and inflammation [50,51]. In addition, in the Amazon and regions in the South of Brazil, hydroalcoholic solutions of *B. pilosa* roots are also regarded as useful in the treatment of malaria [52] and even tumors [53].

Studies of *B. pilosa* plant extracts have shown it has anti-hyperglycemic [54,55], antihypertensive [56,57,58], antiulcerogenic [45], hepatoprotective [59], antipyretic [60], immunosuppressive and anti-inflammatory [8,61,62], anti-leukemic [63,64], anti-malarial [50], anti-bacterial [48], antioxidant [65,66] and antitumor [67] effects. These proven biological activities have led countries like Brazil to include *B. pilosa* in the official list of medicinal plants with potential for development of herbal use by the public health system [68].

Because the biological activities of some extracts and fractions obtained from different parts of *B. pilosa*, several isolated constituents of the plant have been studied, referring to anti-inflammatory activity, immunosuppressive [44,49,61,69,70], hepatoprotective [59], anti-bacterial [44,71], antifungal [71] anti-malarial [50,71,72], anticancer [72], antiparasitic [73], anti-hyperglycemic activities [49,54,70,74,75,76], anti-angiogenic [77,78], antioxidant [79] and cercaricidal [80].

## The Phytochemistry of *Bidens pilosa* L.

*B. pilosa* has been extensively studied since the early 1900s. Among the classes of compounds reported polyacetylenes and flavonoids, typical metabolite classes in the *Bidens* genus, predominate [4,81]. These are also the most reported classes of compounds when referring to the biological activities [49,50,54,61,74,75,82,83]. A number of earlier studies also have reported the isolation of sterols [44,84,85], terpenoids [46,85,86], phenylpropanoids [62,83,87,88,89,90] and hydrocarbons [44,85,91]. 

There have been a few reviews of *B. pilosa* [6,51,92,93], however the phytochemical data have not included all classes of metabolites. To date almost 198 compounds have been described from this species. These secondary metabolites are listed in Table 1, where they were grouped based on the classification adopted by a standard reference work, the Dictionary of Natural Products [94]. 

The order begins with the structurally most simple metabolites, derived from aliphatic natural produts (branched, unbranched, saturated or unsaturated hydrocarbons), and among these, the acetylenes are highlighted. Next the derivatives of simple aromatic hydrocarbons and the phenylpropanoids, in which a C3 substituent is attached to the aromatic unit (C6), form a biosynthetically distinct group of aromatic metabolites. The flavonoids, also considered a large group of metabolites in *B. pilosa* are subdivided into aurones, chalcones, flavanones, flavones and flavonols. The terpenoids group is divided according to the number of carbons, starting in sesquiterpenes and continuing with diterpenes, sterols, triterpenes and finally tetraterpenes. Finally, porphyrins, nitrogen and sulphur-containing natural products, one disaccharide and miscellaneous compounds are arranged.

**Table 1:** Compounds isolated from *Bidens pilosa* L.

N°.	Name	Alternative name	Structure	Plant part	Country	Ref.
***Aliphatic natural products***						
***Saturated unbranched hydrocarbons***						
**1**	heneicosane		CH_3_(CH_2_)_19_CH_3_	AP	Tanzania	[44]
**2**	dodosane		CH_3_(CH_2_)_20_CH_3_	AP	Tanzania	[44]
**3**	tricosane		CH_3_(CH_2_)_21_CH_3_	AP	Tanzania	[44]
**4**	tetracosane		CH_3_(CH_2_)_22_CH_3_	AP	Tanzania	[44]
**5**	pentacosane		CH_3_(CH_2_)_23_CH_3_	AP	Tanzania	[44]
**6**	hexacosane		CH_3_(CH_2_)_24_CH_3_	AP	Tanzania	[44]
**7**	heptacosane		CH_3_(CH_2_)_25_CH_3_	AP	Tanzania	[44]
**8**	octacosane		CH_3_(CH_2_)_26_CH_3_	NF	Taiwan	[91]
				AP	Tanzania	[44]
**9**	nonocosane		CH_3_(CH_2_)_27_CH_3_	NF	Taiwan	[91]
				AP	Tanzania	[44]
**10**	triacontane		CH_3_(CH_2_)_28_CH_3_	NF	Taiwan	[91]
				AP	Tanzania	[44]
**11**	hentriacontane		CH_3_(CH_2_)_29_CH_3_	NF	Taiwan	[91]
				AP	Tanzania	[44]
**12**	dotriacontane		CH_3_(CH_2_)_30_CH_3_	NF	Taiwan	[91]
				AP	Tanzania	[44]
**13**	tritriacontane		CH_3_(CH_2_)_31_CH_3_	NF	Taiwan	[91]
				AP	Tanzania	[44]
***Saturated unbranched alcohols***						
**14**	2-butoxy-ethanol		CH_3_(CH_2_)_3_OCH_2_CH_2_OH	EP	Taiwan	[85]
**15**	tetracosan-1-ol		CH_3_(CH_2_)_22_CH_2_OH	AP	Tanzania	[44]
**16**	hexacosan-1-ol		CH_3_(CH_2_)_24_CH_2_OH	AP	Tanzania	[44]
**17**	1-octacosanol		CH_3_(CH_2_)_26_CH_2_OH	AP	Tanzania	[44]
**18**	1-hentriacontanol		CH_3_(CH_2_)_29_CH_2_OH	NF	Taiwan	[91]
***Saturated unbranched carboxylic acids***						
**19**	tetradecanoic acid	myristic acid	CH_3_(CH_2_)_12_CO_2_H	AP	Tanzania	[44]
**20**	hexadecanoic acid	palmitic acid	CH_3_(CH_2_)_14_CO_2_H	AP	Tanzania	[44]
**21**	octadecanoic acid	stearic acid	CH_3_(CH_2_)_16_CO_2_H	AP	Tanzania	[44]
**22**	eicosanoic acid	arachidic acid	CH_3_(CH2)_18_CO_2_H	AP	Tanzania	[44]
**23**	docosanoid acid	behenic acid	CH_3_(CH2)_20_CO_2_H	LF	not stated	[84]
***Unbranched aliphatic carboxylic acid esters***						
**24**	2-butenedioic acid		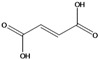	AP	China	[121]
				AP	China	[102]
**25**	(*Z*)-9-octadecenoic acid	oleic acid	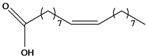	AP	Tanzania	[44]
**26**	(*E*)-9-octadecenoic acid	elaidic acid	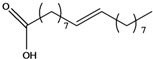	LF	not stated	[84]
**27**	(*Z,Z*)-9,12-octadecadienoic acid	linolic acid/linoleic acid	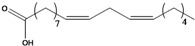	AP	Tanzania	[44]
				EP	Taiwan	[85]
**28**	(*Z,Z,Z*)-9,12,15-octadecatrienoic acid	α-linolenic acid	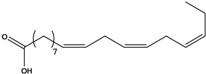	EP	Taiwan	[85]
**29**	(*Z,Z*)-9,12-octadecadienoic acid, ethyl ester	ethyl linoleate	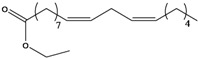	EP	Taiwan	[85]
**30**	(*Z,Z,*Z)-9,12,15-octadecatrienoic acid, methyl ester	methyl linolenate	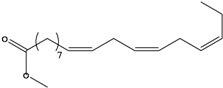	EP	Taiwan	[85]
**31**	(*Z,Z,*Z)-9,12,15-octadecatrienoic acid, ethyl ester	ethyl linolenate	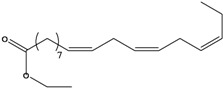	EP	Taiwan	[85]
**32**	(*Z*)-9-octadecenoic acid, 2-butoxyethyl ester	2-butoxyethyl oleate	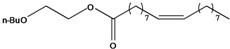	EP	Taiwan	[85]
**33**	2-butoxyethyl linoleate		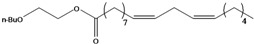	EP	Taiwan	[85]
**34**	(Z,Z,Z)-9,12,15-octadecatrienoic acid, butoxyrthyl ester	2-butoxyethyl linolenate	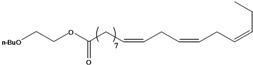	EP	Taiwan	[85]
***Acetylenic hydrocarbons***						
**35**	1,7*E*,9*E*,15*E*-heptadecatetraene-11,13-diyne	heptadeca-2*E*,8*E*,10*E*,16-tetraen-4,6-diyne	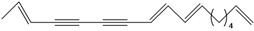	NF	China	[99]
**36**	1,11-tridecadiene-3,5,7,9-tetrayne		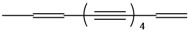	RT	not stated	[2]
**37**	1-tridecaene-3,5,7,9,11-pentayne	pentayneene	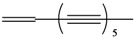	LF	not stated	[2]
				NF	Egypt	[86]
**38**	5-tridecaene-7,9,11-triyne-3-ol		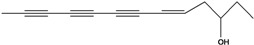	NF	Egypt	[86]
**39**	2,10,12-tridecatriene-4,6,8-triyn-1-ol		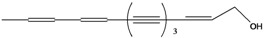	PNS	not stated	[51]
**40**	2,12-tridecadiene-4,6,8,10-tetrayn-1-ol	1,11-tridecadiene-3,5,7,9-tetrayn-13-ol	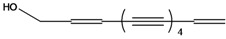	RT	not stated	[2]
				NF	Egypt	[86]
**41**	2,12-tridecadiene-4,6,8,10-tetraynal	1,11-tridecadiene-3,5,7,9-tetrayne-13-al	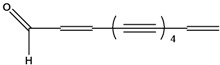	RT	Germany	[122]
**42**	2,12-tridecadiene-4,6,8,10-tetrayn-1-ol,1-acetate	1,11-tridecadiene-3,5,7,9-tetrayne-13-acetate	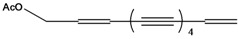	RT	not stated	[2]
**43**	(5*E*)-1,5-tridecadiene-7,9-diyn-3,4,12-triol		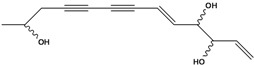	AP	China	[100]
**44**	(6*E*,12*E*)-3-oxo-tetradeca-6,12-dien-8,10-diyn-1-ol		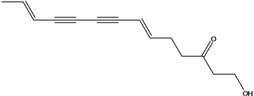	AP	China	[100]
**45**	(E)-5-tridecene-7,9,11-triyne-1,2-diol	1,2-dihydroxy-5(*E*)-tridecene-7,9,11-triyne	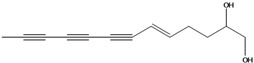	EP	Taiwan	[78]
**46**	(E)-6-tetradecene-8,10,12-triyne-1,3-diol	1,3-dihydroxy-6(*E*)-tetradecene-8,10,12-triyne	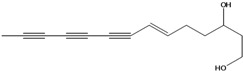	EP	Taiwan	[77]
				EP	Taiwan	[65]
				EP	Taiwan	[78]
**47**	(2*R*,3*E*,11*E*)-3,11-tridecadiene-5,7,9-triyne-1,2-diol	safynol	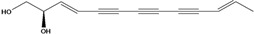	NF	Egypt	[86]
				NF	China	[99]
**48**	5,7,9,11-tridecatetrayne-1,2-diol	1,2-dihydroxy-trideca-5,7,9,11-tetrayne	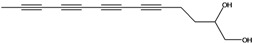	EP	Taiwan	[77]
				EP	Taiwan	[78]
**49**	(R)-3,5,7,9,11-tridecapentayne-1,2-diol	(*R*)-1,2-dihydroxy-trideca-3,5,7,9,11-pentayne	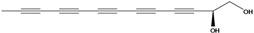	AP	Japan	[71]
**50**	(4E)-1-(hydroxyl-methyl)-4-dodecene-6,8,10-triyn-1-yl-β-D-glucopyranoside	2-*β*-*D*-gluco-pyranosyloxy-1-hydroxy-5(*E*)-tridecene-7,9,11-triyne	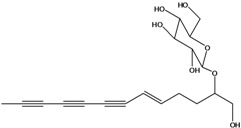	AP	USA	[54]
				EP	Taiwan	[75]
				EP	Taiwan	[123]
				EP	Taiwan	[65]
				EP	Taiwan	[49]
				LF	Taiwan	[124]
**51**	(4*E*)-1-(2-hydroxy-ethyl)-4-dodecene-6,8,10-triyn-1-yl-*β-D*-glucopyranoside	3-*β*-*D*-gluco-pyranosyloxy-1-hydroxy-6(*E*)-tetradecene-8,10,12-triyne	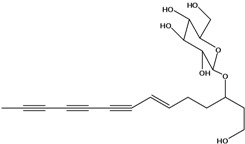	AP	USA	[54]
				AP	China	[102]
				EP	Taiwan	[75]
				EP	Taiwan	[123]
				EP	Taiwan	[65]
				EP	Taiwan	[49]
				LF	Taiwan	[124]
				AP	China	[100]
**52**	3-hydroxy-6-tetra-decene-8,10,12-triynyl-*β*-*D*-gluco-pyranoside	*β*-*D*-gluco-pyranosyloxy-3-hydroxy-6*E*-tetradecene-8,10,12-triyne	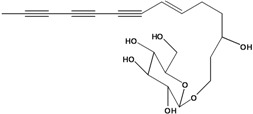	EP	Mexico	[53]
**53**	1-(hydroxymethyl)-4,6,8,10-dodeca-tetrayn-1-yl-*β-D*-glucopyranoside	2-*β*-*D*-gluco-pyranosyloxy-1-hydroxytrideca-5,7,9,11-tetrayne , cytopiloyne	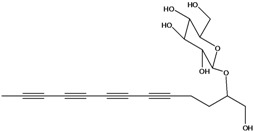	EP	Taiwan	[49]
				EP	not stated	[82]
				LF	Taiwan	[124]
**54**	2-*O*-*D*-glucosyltrideca-11*E*-en-3,5,7,9-tetrayn-1,2-diol		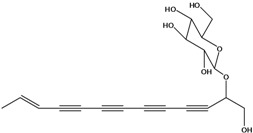	LF	Brazil	[61]
**55**	(*R*)-1-(hydroxy-methyl)-2,4,6,8,10-dodecapentayn-1-yl-*β-D*-glucopyranoside	2-*β*-*D*-gluco-pyranosyloxy-1-hydroxytrideca-3,5,7,9,11-pentayne	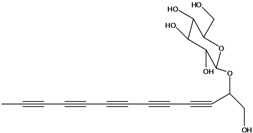	AP	China	[102]
				AP	Japan	[71]
**56**	1-[[(carboxy-acetyl)oxy]methyl]-4,6,8,10-dodeca-tetraynyl-*β-D*-glucopyranoside		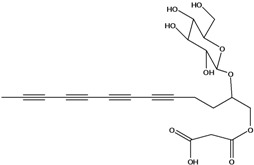	AP	Japan	[125]
**57**	(4*E*)-1-[[(carboxy-acetyl)oxy]-methyl]-4-dodecene-6,8,10-triynyl-*β-D*-gluco-pyranoside		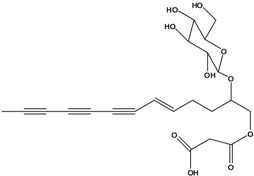	AP	Japan	[125]
**58**	(4*E*)-1-[[(carboxy-acetyl)oxy]-ethyl]-4-dodecene-6,8,10-triynyl-*β-D*-gluco-pyranoside		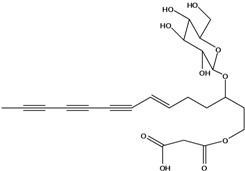	AP	Japan	[125]
**59**	(5*E*)-5-heptene-1,3-diyn-1-yl-benzene	1-phenylhepta-1,3-diyn-5-en	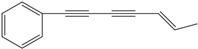	EP	Taiwan	[85]
**60**	7-phenyl-2(*E*)-heptene-4,6-diyn-1-ol		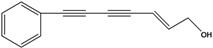	RT	not stated	[2]
				AP	China	[100]
**61**	7-phenyl-2(*E*)-heptene-4,6-diyn-1-ol-acetate		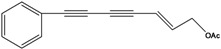	RT	not stated	[2]
				RT	Brazil	[50]
				RT	Brazil	[52]
**62**	7-phenyl-4,6-heptadiyn-2-ol	(-)-pilosol A	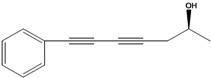	EPAP	TaiwanChina	[85]
						[100]
**63**	7-phenylhepta-4,6-diyn-1,2-diol		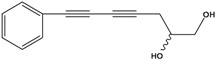	AP	China	[100]
**64**	1,3,5-heptatriyn-1-yl-benzene	1-phenylhepta-1,3,5-triyne	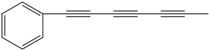	LF	not stated	[2]
				LTC	not stated	[97]
				AP	Tanzania	[44]
				AP	China	[121]
				EP	Taiwan	[85]
				RT	Brazil	[52]
				AP	China	[100]
**65**	7-phenyl-2,4,6-heptatriyn-1-ol		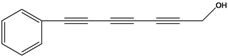	LF	not stated	[2]
				AP	China	[100]
**66**	7-phenyl-2,4,6-heptatriyn-1-ol-acetate		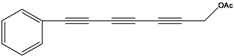	LF	not stated	[2]
**67**	5-(2-phenylethynyl)-2-thiophene methanol		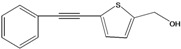	AP	China	[100]
**68**	5-(2-phenylethynyl)-2β-glucosylmethyl-thiophene		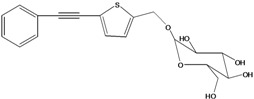	AP	China	[100]
***Simple aromatic hydrocarbons***						
***Simple phenols***						
**69**	1,2-benzenediol	pyrocatechin	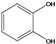	EP	Japan	[87]
**70**	4-ethyl-1,2-benzenediol	pyrocatechol	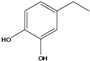	EP	Japan	[87]
**71**	dimethoxyphenol		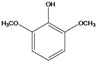	RT	Japan	[87]
**72**	4-ethenyl-2-methoxy-phenol	*p*-vinylguaiacol	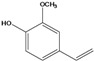	EP	Japan	[87]
**73**	2-hydroxy-6-methylbenzaldehyde	6-methyl-salicylaldehyde	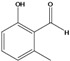	EP	Japan	[87]
**74**	benzene-ethanol	2-phenyl-ethanol	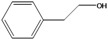	EP	Taiwan	[85]
***Simple aryl aldehydes***						
**75**	4-hydroxy-3-methoxy-benzaldehyde	vanillin	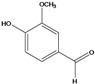	AP	Japan	[87]
**76**	3-hydroxy-4-methoxy-benzaldehyde	vanillin, *iso*	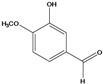	LF	Japan	[87]
***Simple benzoic acids and their homologues***						
**77**	4-hydroxy-benzoic acid	*p*-hydroxybenzoic acid	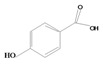	EP	Japan	[87]
**78**	2-hydroxy-benzoic acid	salicylic acid	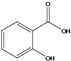	ST/RT	Japan	[87]
**79**	3,4-dihydroxy-benzoic acid	protocatechuic acid	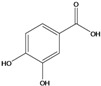	EP	Japan	[87]
**80**	4-hydroxy-3-methoxy-benzoic acid	vanillic acid	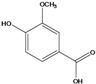	AP	Uganda	[110]
				RT	Japan	[87]
**81**	3,4,5-trihydroxy-benzoic acid	gallic acid	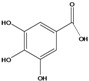	EP	China	[126]
***Phenylpropanoids***						
***Simple phenylpropanoids***						
**82**	3-(4-hydroxyphenyl)-2-propenoic acid	*p*-coumaric acid	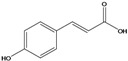	EP	Japan	[87]
**83**	2-methoxy-4(2-propen-1-yl)-phenol	eugenol	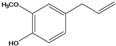	LF/RT	Japan	[87]
**84**	3-(4-hydroxy-3-methoxyphenyl)-2propenoic acid	ferulic acid	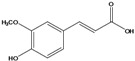	EP	Japan	[87]
**85**	3-(3,4-dihydroxy-phenyl)-2-propenoic acid	caffeic acid	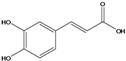	EP	Japan	[87]
				AP	Japan	[62]
**86**	3-propyl-3-[(2,4,5-trimetoxyphenyl)-methoxy]-2,4-pentanedione	3-propyl-3-(2,4,5-trimethoxy)benzyloxy-pentan-2,4-dione	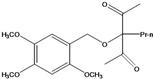	LF	India	[90]
***Coumaric and caffeoyl esters***						
**87**	3-(3,4-dihydroxy-phenyl)-2-propenoic acid, ethyl ester	caffeate, ethyl	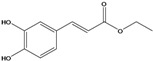	NF	Taiwan	[127]
				EP	Taiwan	[65]
				EP	Taiwan	[78]
**88**	2-[[3-(3,4-dihydroxy-phenyl)-1-oxo-2-propenyl]oxy]-3,4-dihydroxy-2-methyl-butanoic acid	*d*-erythronic acid, 2-*O*-caffeoyl-2-*C*-methyl	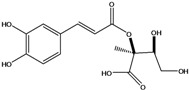	LF	Japan	[88]
**89**	2-[[3-(3,4-dihydroxy-phenyl)-1-oxo-2-propenyl]oxy]-3,4-dihydroxy-2-methyl-butanoic acid,methyl ester	*d*-erythronate, methyl 2-*O*-caffeoyl-2-*C*-methyl	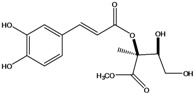	LF	Japan	[88]
**90**	3-[[3-(3,4-dihydroxy-phenyl)-1-oxo-2-propenyl]oxy]-2,4-dihydroxy-2-methyl-butanoic acid,methyl ester	*d*-erythronate, methyl 3-*O*-caffeoyl-2-*C*-methyl	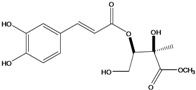	LF	Japan	[88]
**91**	4-(acetyloxy)-3-[[3-(3,4-dihydroxyphenyl)-1-oxo-2-propen-1-yl]oxy]-2-hydroxy-2-methyl-butanoic acid		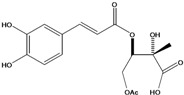	NF	Japan	[70]
**92**	3-(3,4-dihydroxyphenyl)- tetrahydro-4-hydroxy-4-methyl-5-oxo-3-furanyl ester-2 propenoic acid	3-*O*-caffeoyl-2-*C*-methyl-*D*-erythrono-1,4-lactone	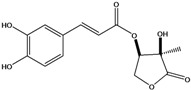	LF	Japan	[88]
**93**	3-[[3-(3,4-dihydroxyphenyl)-1-oxo-2-propen-1-yl]oxy]-1,4,5-trihydroxy-cyclo-hexanecarboxylic acid	chlorogenic acid	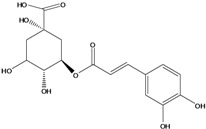	AP	Japan	[83]
				EP	Taiwan	[79]
				AP	Japan	[62]
**94**	4-[[3-(3,4-dihydroxy-phenyl)-1-oxo-2-propen-1-yl]-oxy]-1,3,5-trihydroxy-cyclo-hexanecarboxylic acid	4-*O*-caffeoylquinic acid	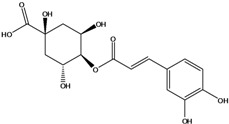	AP	Japan	[83]
**95**	3,4-bis[[(2*E*)-3-(3,4-dihydroxyphenyl)-1-oxo-2-propen-1-yl]-oxy]-1,5-dihydroxy-cyclohexane--carboxylic acid	3,4-di-*O*-caffeoylquinic acid	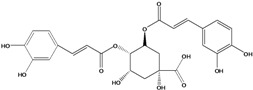	AP	Japan	[83]
				EP	Taiwan	[79]
				EP	Taiwan	[75]
				EP	Taiwan	[65]
**96**	3,5-bis[[(2*E*)-3-(3,4-dihydroxyphenyl)-1-oxo-2-propen-1-yl]-oxy]-1,4-dihydroxy-cyclohexane-carboxylic acid	3,5-di*-O-*caffeoylquinic acid	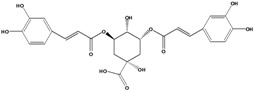	AP	Japan	[83]
				EP	Taiwan	[79]
				EP	Taiwan	[75]
				EP	Taiwan	[65]
**97**	3,4-bis[[(2*E*)-3-(3,4-dihydroxyphenyl)-1-oxo-2-propen-1-yl]-oxy]-1,5-dihydroxy-cyclohexane-carboxylic acid	4,5-di*-O-*caffeoylquinic acid	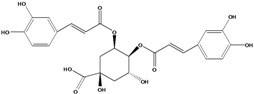	EP	Taiwan	[79]
				EP	Taiwan	[75]
				EP	Taiwan	[65]
**98**	3-[4-[[6-O-[3-(4-hydroxyphenyl)-1-oxo-2-propen-1-yl]-*β-D*-glucopyranosyl]-oxy]-phenyl]-2-propenoic acid	*β*-*D*-*p*-coumaric acid, 4-*O*-(6-*O*-*p*-coumaroyl-glucopyranosyl)	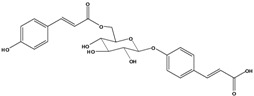	LF	Japan	[89]
**99**	3-[4-[[2-O-acetyl-6-O-[3-(4-hydroxyphenyl)-1-oxo-2-propen-1-yl]-*β-D-*glucopyranosyl]-oxy]-phenyl]-2-propenoic acid	*β*-*D-p*-coumaric acid, 4-*O-*(2-*O*-acetyl-6-*O*-*p*-coumaroyl-glucopyranosyl)	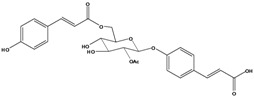	LF	Japan	[89]
				AP	China	[121]
***Coumarins***						
**100**	6,7-dihydroxy-2H-1-benzopyran-4-one	esculetin	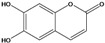	NF	Egypt	[86]
***Flavonoids***						
***Aurones***						
**101**	2-[(3,4-dihydroxy-phenyl)-methylene]-6-hydroxy-3(2H)-benzofuranone	sulfuretin	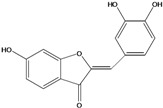	AP	China	[102]
**102**	2-[(3,4-dihydroxy-phenyl)-methylene]-6,7-dihydroxy-3(2H)-benzofuranone	aurone, (*Z*)-6,7,3’,4’-tetrahydroxy; maritimetin	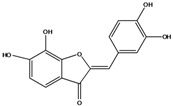	AP	China	[102]
**103**	2-[(3,4-dihydroxy-phenyl)-methylene]-6-(*β-D*-glucopyranos-yloxy)-7-hydroxy-3(2H)-benzofuranone	aurone, (*Z*)-6-*O-β-D*-glucopyranosyl-6,7,3',4'-tetrahydroxy; maritimein	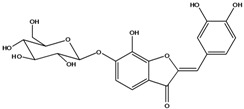	LFAPLF	Japan	[89]
					China	[102]
					China	[59]
**104**	2-[(3,4-dihydroxy-phenyl)-methylene]-7-(*β-D-*glucopyranos-yloxy)-6-hydroxy-3(2H)-benzofuranone	aurone, (*Z*)-7-*O*-*β-D*-glucopyranosyl-6,7,3',4'-tetrahydroxy	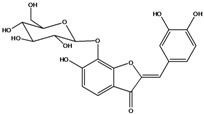	LF	Japan	[89]
**105**	6-[(6-*O*-acetyl-*β-D*-glucopyranosyl)oxy]-2-[(3,4-dihydroxy-phenyl)-methylene]-7-hydroxy-3(2H)-benzofuranone	aurone, (*Z*)-6-*O-*( 6-*O*-acetyl-*β-D*-glucopyranosyl)-6,7,3’,4’-tetrahydroxy	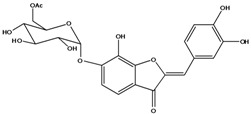	LF AP	Japan	[89]
					China	[102]
**106**	6-[(3,6-di-*O*-acetyl*-β-D-*glucopyranosyl)-oxy]-2-[(3,4-di-hydroxyphenyl)-methylene]-7-hydroxy-3(2H)-benzofuranone	aurone, (*Z*)-6-*O*-(3,6-di-*O*-acetyl-*D*-glucopyranosyl)-6,7,3’,4’-tetrahydroxy; bidenoside A	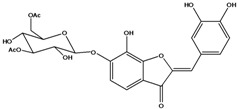	LF	China	[59]
**107**	6-[(4,6-di-*O*-acetyl-*β-D-*glucopyranosyl)-oxy]-2-[(3,4-di-hydroxyphenyl)-methylene]-7-hydroxy-3(2H)-benzofuranone	aurone, (*Z*)-6-*O-*(4”,6”-diacetyl-*β-D*-glucopyranosyl)-6,7,3’,4’-tetrahydroxy	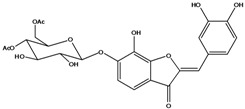	LF	not stated	[128]
				AP	China	[121]
				AP	China	[102]
**108**	2-[(3,4-dihydroxy-phenyl)-methylene]-7-hydroxy-6-[(2,4,6-tri-*O*-acetyl-*β-D*-gluco-pyranosyl)-oxy-3(2H)-benzofuranone]	aurone, (*Z*)-6-*O*-(2”,4”,6”-triacetyl-*β-D*-glucopyranosyl)-6,7,3’,4’-tetrahydroxy	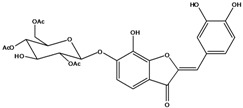	LF	not stated	[128]
				AP	China	[121]
**109**	2-[(3,4-dihydroxy-phenyl)-methylene]-7-hydroxy-6-[(3,4,6-tri-*O*-acetyl-*β-D*-gluco-pyranosyl)-oxy]-3(2H)-benzofuranone	aurone, (*Z*)-6-*O-*(3”,4”,6”-triacetyl-*β-D*-glucopyranosyl)-6,7,3’,4’-tetrahydroxy	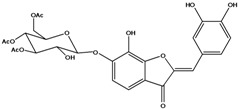	AP	China	[121]
				AP	China	[102]
**110**	2-[(3,4-dihydroxy-phenyl)-methylene]-7-hydroxy-6-[[6-O-[3-(4-hydroxyphenyl)-1-oxo-2-propenyl]-*β-D*-glucopyranosyl]oxy]-3(2H)-benzofuranone	aurone, (*Z*)-6-*O*-(6-*O*-*p*-coumaroyl-*β-D*-glucopyranosyl)-6,7,3',4'-tetrahydroxy	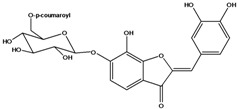	LF	Japan	[89]
***Chalcones***						
**111**	1-[2-(β-D-gluco-pyranosyloxy)-4-hydroxyphenyl]-2-hydroxy-3-(3-hydroxyphenyl)- 2-propen-1-one	chalcone, *α*,3,2’,4’-tetrahydroxy-2’-*O*-*β-D*-glucopyranosyl	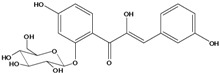	AP	China	[102]
**112**	1-(2,4-dihydroxy-phenyl)-3-(3,4-dihydroxy-phenyl)-2-propen-1-one	butein	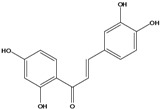	AP	China	[102]
**113**	3-(3,4-dihydroxy-phenyl)-1-(2,3,4-trihydroxy-phenyl)-2-propen-1-one	okanin	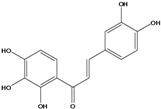	LF	China	[59]
**114**	3-(3,4-dihydroxy-phenyl)-1-[3-(β-D-glucopyranosyloxy)-2,4-dihydroxyphenyl]-2-propen-1-one	okanin 3’-*O*-*β-D*-glucoside	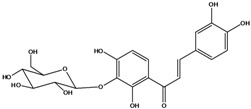	LF	Germany	[129]
				LF	Germany	[130]
				FL	Germany	[109]
**115**	3-(3,4-dihydroxy-phenyl)-1-[4-(β-D-glucopyranosyloxy)-2,3-dihydroxyphenyl]-2-propen-1-one	okanin 4’-*O*-*β-D*-glucopyranoside; marein	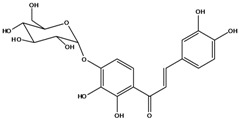	FL	Germany	[109]
				LF	Japan	[89]
**116**	okanin 4’-*O*-*β-D*-(6”-*O*-acetylglucoside)		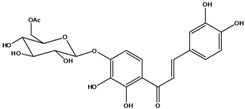	FL	Germany	[109]
**117**	1-[4-[(4,6-di-O-acetyl-β-D-glucopyranosyl)-oxy]-2,3-dihydroxy-phenyl]-3-(3,4-di-hydroxyphenyl)-2-propen-1-one	okanin 4’-*O-β-D*-(4”,6”-diacetyl)-glucopyranoside	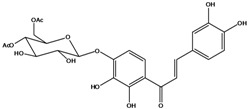	AP	China	[121]
**118**	okanin 4’-*O*-*β-D*-(2”,4”,6”-triacetyl)-glucoside		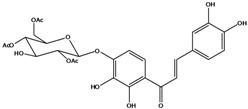	LF	Germany	[129]
**119**	okanin 4’-*O-β-D*-(3”,4”,6”-triacetyl)-glucoside		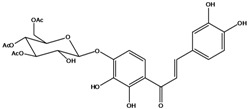	AP	China	[121]
**120**	1-[2,3-dihydroxy-4-[[6-O-[3-(4-hydroxy-phenyl)-1-oxo-2-propenyl]-*β-D*-glucopyranosyl]oxy]-phenyl]-3-(3,4-dihydroxyphenyl)-2-propen-1-one	okanin 4’-*O*-*β-D*-(6”-*trans-p*-coumaroyl) glucoside	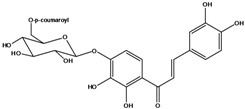	LF	Germany	[129]
**121**	okanin 4’-*O*-*β-D*-(4”-acetyl-6”-*trans-p*-coumaroyl)-glucoside		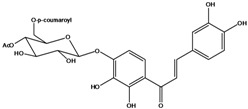	LF	Germany	[131]
**122**	okanin 4’-*O*-*β-D*-(2”,4”-diacetyl-6”-*trans-p*-coumaroyl)-glucoside		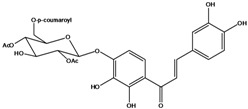	LF	Germany	[131]
**123**	okanin 4’-*O*-*β-D*-(3”,4”-diacetyl-6”-*trans-p*-coumaroyl)-glucopyranoside		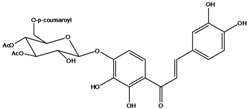	LF	Germany	[131]
**124**	okanin 4’-*O-*[*β-D*-glucopyranosyl-(1→6)-*β-D*-glucopyranoside]		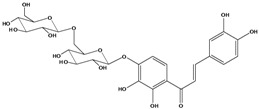	FL	Germany	[109]
**125**	okanin 3’,4’-di-*O*-*β-D*-glucoside		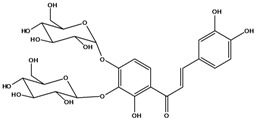	FL	Germany	[109]
**126**	1-[3-(β-D-gluco-pyranosyloxy)-2,4-dihydroxyphenyl]-3-(3-hydroxy-4-methoxyphenyl)-2-propen-1-one	okanin 4-methyl ether-3’-*O*-*β-D*-glucopyranoside	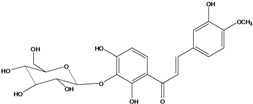	LF	Germany	[130]
				AP	China	[102]
**127**	okanin 4-methyl ether-3’,4’-di-*O-β*-(4”,6”,4’’’,6’’’-tetracetyl)-glucopyranoside		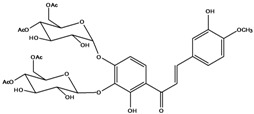	AP	China	[100]
**128**	chalcone, 2’,4’,6’-trimethoxy-4-*O*-*D*-glucopyranosyl-dihydro		NF	LF	China	[59]
***Flavanones***						
**129**	2-(3,4-dihydroxy-phenyl)-2,3-dihydro-7,8-dihydroxy-4H-1-benzopyran-4-one	okanin,*iso*	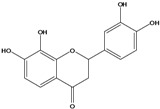	LF	China	[59]
**130**	2-(3,4-dihydroxy-phenyl)-2,3-dihydro-8-hydroxy-7-[(2,4,6-tri-*O*-acetyl-*β-D*-gluco-pyranosyl)oxy]-4H-1-benzopyran-4-one	okanin 7-*O-β-D*-(2”,4”,6”-triacetyl)-glucopyranoside, *iso*	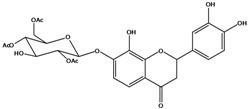	AP	China	[121]
***Flavones***						
**131**	5,7-dihydroxy-2-(4-hydroxyphenyl)- 4H-1-benzopyran-4-one	apigenin	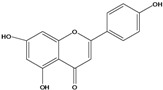	AP	Tanzania	[44]
				AP	China	[100]
**132**	7-(*β-D*-glucopyranos-yloxy)-5-hydroxy-2-(4-hydroxyphenyl)-4H-1-benzopyran-4-one	apigenin 7-*O*-glucopyranoside	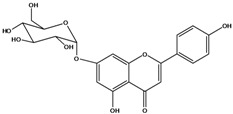	AP	Tanzania	[44]
**133**	2-(3,4-dihydroxy-phenyl)-5,7-dihydroxy-4H-1-benzopyran-4-one	luteolin	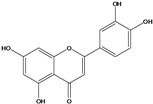	AP	Tanzania	[44]
				AP	China	[121]
				AP	China	[102]
				AP	China	[100]
				AP	Vietnam	[132]
**134**	2-(3,4-dihydroxy-phenyl)-7-(*β-D*-gluco-pyranosyloxy)-5-hydroxy-4H-1-benzopyran-4-one	luteolin 7-*O-β-D*-glucopyranoside	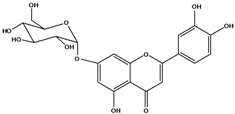	AP	Tanzania	[44]
**135**	5,7-dimethoxy-6-(5-methoxy-6-methyl-4-oxo-4H-pyran-3-yl)-2-phenyl-4H-1-benzopyran-4-one	5-*O-*methylhoslundin	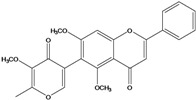	AP	Uganda	[110]
***Flavonols***						
**136**	3-(*β-D*-gluco-pyranosyloxy)-5,7-dihydroxy-2-(4-hydroxyphenyl)- 4H-1-benzopyran-4-one	astragalin; kaempferol-3-*O-β-D-*glucopyranoside	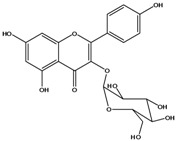	AP	China	[102]
**137**	kaempferol 3-(2,3-di-*E-p*-coumaroyl-*α-L*-rhamnopyranoside)		NF	AP	Vietnam	[132]
**138**	2-(3,4-dihydroxy-phenyl)-7-(*β-D*-glucopyranosyloxy)-5-hydroxy-3,6-dimethoxy-4H-1-benzopyran-4-one	axillaroside	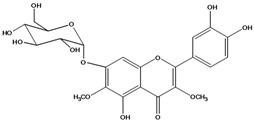	AP	China	[100]
**139**	5,7-dihydroxy-2-(3-hydroxy-4-methoxy-phenyl)-3,6-di-methoxy-4H-1-benzopyran-4-one	centaureidin	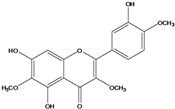	EP	Taiwan	[74]
**140**	7-(*β-D*-glucopyranos-yloxy)-5-hydroxy-2-(3-hydroxy-4-methoxyphenyl)-3,6-dimethoxy-4H-1-benzopyran-4-one	centaurein	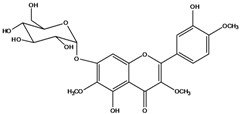	AP	Japan	[83]
				EP	Taiwan	[79]
				EP	Taiwan	[74]
**141**		eupatorin, *iso*	NF	NF	China	[99]
**142**	2-(3,4-dimethoxy-phenyl)-7-(*β-D*-glucopyranosyloxy)-3,5-dihydroxy-8-methoxy-4H-1-benzopyran-4-one		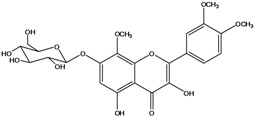	NF	Japan	[70]
**143**	7-(*β-D*-glucopyranos-yloxy)-5-hydroxy-2-(4-hydroxy-3-methoxyphenyl)-3,8-dimethoxy-4H-1-benzopyran-4-one		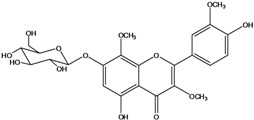	NF	Japan	[70]
**144**		isorhamnetin 3-[*O*-*α-L-*rhamno-pyranosyl-(1-2)-*β-D*-glucopyranoside]	NF	AP	Vietnam	[132]
**145**	7-[(6-deoxy-*α-L*-mannopyranosyl)oxy]-3-(*β-D*-glucopyranos-yloxy)-5-hydroxy-2-(4-hydroxy-3-methoxyphenyl)-4H-1-benzopyran-4-one	luteoside	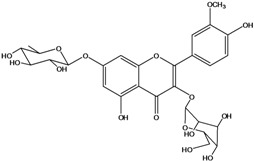	AP	China	[100]
**146**	luteolin 3-*O-β-D*-glucopyranoside		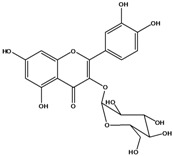	AP	Tanzania	[44]
**147**	5,7-dihydroxy-2-(4-hydroxy-3-methoxy-phenyl)-3,6-di-methoxy-4H-1-benzopyran-4-one	quercetagetin 3,6,3′-trimethyl ether	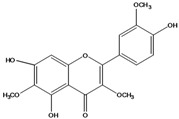	AP	China	[100]
**148**		quercetagetin 3,7,3’-trimethyl ether-6-*O*-*β*-glucoside	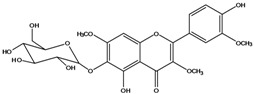	AP	China	[100]
**149**	7-(*β-D*-glucopyranos-yloxy)-5-hydroxy-2-(4-hydroxy-3-methoxyphenyl)-3,6-dimethoxy-4H-1-benzopyran-4-one	jacein; quercetagetin 3,6,3′-trimethyl ether-7-*O-β*-glucoside	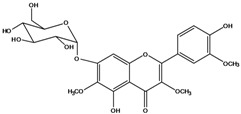	AP	Japan	[83]
				EP	Taiwan	[79]
				AP	China	[100]
**150**	2-(3,4-dihydroxy-phenyl)-3,5,7-trihydroxy- 4H-1-benzopyran-4-one	quercetin	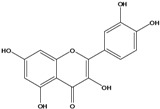	AP	China	[102]
				LF	China	[59]
				EP	China	[133]
**151**	2-(3,4-dihydroxy-phenyl)-3-(*β-D*-galactopyranosyloxy)-5,7-dihydroxy-4H-1-benzopyran-4-one	quercetin 3-*O-β-D*-galactoside; hyperin; hyperoside	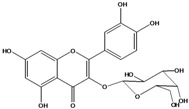	AP	Tanzania	[44]
				AP	Japan	[83]
				NF	China	[99]
				AP	Japan	[62]
				LF	China	[59]
				EP	China	[133]
**152**	2-(3,4-dihydroxy-phenyl)-3-(*β-D*-glucopyranosyloxy)-5,7-dihydroxy-4H-1-benzopyran-4-one	quercetin 3-*O-β-D*-glucopyranoside	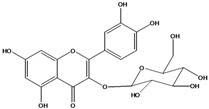	AP	Tanzania	[44]
				LF	Japan	[89]
				AP	China	[102]
				AP	Japan	[62]
**153**	2-(3,4-dihydroxy-phenyl)-5,7-dihydroxy-4-oxo-4H-1-benzo-pyran-3-yl-*β-D*-glucopyranosiduronic acid	quercetin *3-O-β-D-*glucuronopyranoside	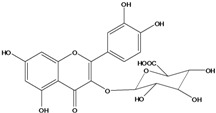	AP	Tanzania	[44]
				AP	Japan	[83]
**154**	3-[[6-O-(6-deoxy-α-L-mannopyranosyl)-β-D-galactopyranosyl]oxy]-2-(3,4-dihydroxy-phenyl)-5,7-dihydroxy- 4H-1-benzopyran-4-one	quercetin 3-O-robinobioside	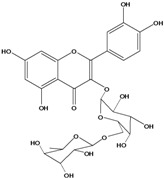	AP	Japan	[83]
				EP	Taiwan	[79]
**156**	7-(*β-D*-glucopyranos-yloxy)-5-hydroxy-2-(4-hydroxy-3-methoxyphenyl)-3-methoxy-4H-1-benzopyran-4-one	quercetin 3,3’-dimethyl ether 7*-O-β*-*D*-glucopyranoside	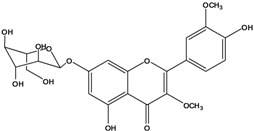	RT	Brazil	[134]
				RT	Brazil	[52]
				RT	Brazil	[135]
**157**	7-[[6-O-(6-deoxy-α-L-mannopyranosyl)-β-D-glucopyranosyl]oxy]-5-hydroxy-2-(4-hydroxy-3-methoxy-phenyl)-3-methoxy-4H-1-benzopyran-4-one	quercetin 3,3’-dimethyl ether 7-*O-α*-*L*-rhamnopyranosyl-(1→6)-*β*-*D*-glucopyranoside	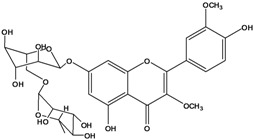	RT	Brazil	[134]
				RT	Brazil	[52]
**158**	7-[[6-O-(6-deoxy-α-L-mannopyranosyl)-β-D-glucopyranosyl]oxy]-5-hydroxy-2-(3-hydroxy-4-methoxy-phenyl)-3-methoxy-4H-1-benzopyran-4-one	quercetin 3,4’-dimethyl ether-7*-O-*rutinoside	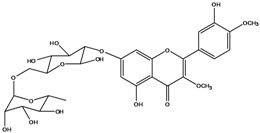	AP	China	[121]
				AP	China	[102]
**159**	2-(3,4-dihydroxy-phenyl)-3-(*β-D*-glucofuranosyloxy)-5,7-dihydroxy-4H-1-benzopyran-4-one	isoquercitrin	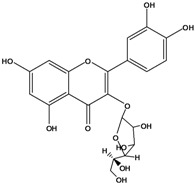	AP	Japan	[83]
				AP	China	[102]
***Terpenoids***						
***Sesquiterpenes***						
**160**	3,7,11,11-tetramethyl-bicyclo[8.1.0]undeca-2,6-diene	bicyclogermacrene	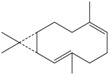	LF	Brazil	[46]
**161**	4,11,11-trimethyl-8-methylenebicyclo-[7.2.0]undec-4-ene	*E*-caryophyllene	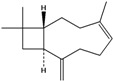	LF	Brazil	[46]
**162**	1-methyl-5-methylene-8-(1-methylethyl)-1,6-cyclodecadiene	germacrene-D	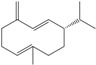	LF	Brazil	[46]
**163**	4-(1,5-dimethyl-4-hexen-1-ylidene)-1-methyl-cyclohexene	*Z-γ-*bisabolene	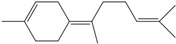	LF	Brazil	[46]
**164**	decahydro-1,1,4-trimethyl-7-methylene-1H-cycloprop[e]-azulene	*β*-gurjunene	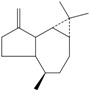	LF	Brazil	[46]
**165**	2,6,6,9-tetramethyl-1,4,8-cycloundeca-triene	*α*-humulene;α-caryophyllene	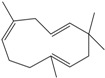	LF	Brazil	[46]
**166**		*δ*-muurolene	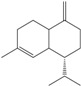	LF	Brazil	[46]
**167**	1,2,3,4,4a,5,6,8a-octahydro-4a,8-dimethyl-2-(1-methylethylidene)-naphthalene	selina-3,7(11)-diene	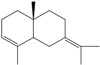	LF	Brazil	[46]
***Diterpenes***						
**168**	(2*E*,7*R*,11*R*)-3,7,11,15-tetramethyl-2-hexadecen-1-ol	phytol	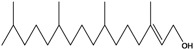	EP	Taiwan	[85]
**169**	3,7,11,15-tetramethyl-2-hexadecenoic acid	phytenic acid	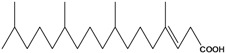	EP	Taiwan	[85]
**170**	3,7,11,15-tetramethyl-2-hexadecenyl ester-heptanoic acid	phythyl heptanoate	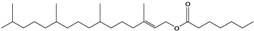	LF	not stated	[84]
***Steroids***						
**171**		campestrol	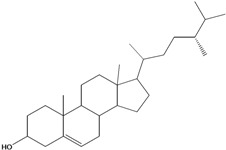	AP	Tanzania	[44]
**172**		phytosterin-B	NF	NF	Taiwan	[112]
				NF	Egypt	[86]
**173**	stigmast-5-en-3-ol	*β*-sitosterol	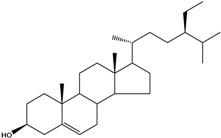	NF	Taiwan	[91]
				AP	Tanzania	[44]
				EP	Taiwan	[85]
**174**		*β*-sitosterol glucoside	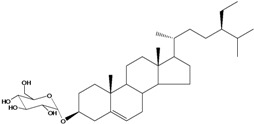	NF	Egypt	[86]
**175**	5*α*-stigmasta-7-en-3*β*-ol		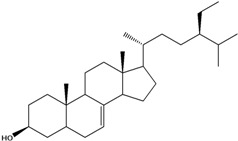	EP	Taiwan	[85]
**176**	5*α*-stigmasta-7,22t-dien-3*β*-ol		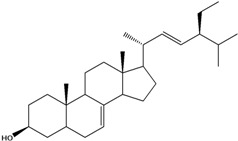	EP	Taiwan	[85]
**177**	stigmasta-5,22-dien-3-ol	stigmasterol	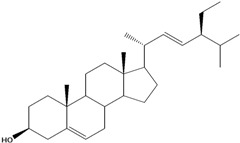	NF	Taiwan	[91]
				AP	Tanzania	[44]
				LF	not stated	[84]
				EP	Taiwan	[85]
***Triterpenes***						
**178**	lup-20(29)-en-3-ol	lupeol	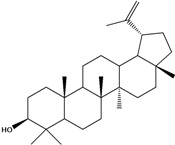	NF	Egypt	[86]
**179**	lup-20(29)-en-3-ol, acetate	lupeol acetate	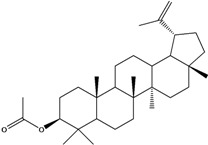	NF	Egypt	[86]
**180**	olean-12-en-3-ol	*β*-amirin	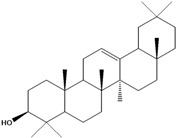	NF	Egypt	[86]
**181**	5,9,13-trimethyl-24,25,26-trinoroleanan-3-ol	friedelan-3*β*-ol	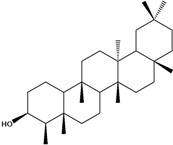	AP	Tanzania	[44]
**182**	5,9,13-trimethyl-24,25,26-tri-noroleanan-3-one	friedelin; friedelan-3-one	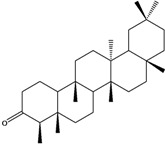	AP	Tanzania	[44]
**183**	2,6,10,15,19,23-hexamethyl-2,6,10,14,18,22-tetracosahexaene	squalene	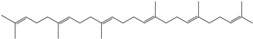	AP	Tanzania	[44]
				LF	not stated	[84]
				EP	Taiwan	[85]
***Tetraterpenes***						
**184**	*β,β*-carotene	*β*-carotene	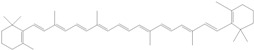	LF	not stated	[113]
***Porphyrins***						
**185**	(2*E*,7*R*,11*R*)-3,7,11,15-tetramethyl-2-hexa-decen-1-yl ester-(15*S*,16*S*)-10-ethenyl-5-ethyl-1,16,18,20-tetrahydro-6,11,15,22-tetramethyl-18,20-dioxo-15H-9,12-imino-21,2-metheno-4,7:17,14-dinitrilo-pyrano[4,3-b]azacyclo-nonadecine-16-propanoic acid	aristophyll-C	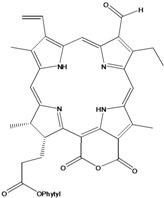	LF	Taiwan	[90]
**186**	(2*E*,7*R*,11*R*)-3,7,11,15-tetramethyl-2-hexadecen-1-yl ester-(2*S*,18*S*,19*S*,20b*R*)-13-ethenyl-8-ethyl-2a,18,19,20b-tetrahydro-20b-(methoxycarbonyl)-9,14,18,24-tetra-methyl-4H-12,15-imino-3,5-metheno-7,10:20,17-dinitrilo-1,2-dioxeto-[3',4':3,4]-cyclo-pent[1,2b]aza-cyclo-nonadecine-19-propanoic acid	bidenphytin A	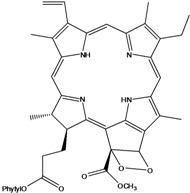	LF	Taiwan	[90]
**187**	(2*E*,7*R*,11*R*)-3,7,11,15-tetramethyl-2-hexa-decen-1-yl ester-(2*S*,18*S*,19*S*,20bR)-13-ethenyl-8-ethyl-2a,18,19,20b-tetrahydro-2a-hydroxy-20b-(methoxy-carbonyl)-9,14,18,24-tetramethyl-4H-12,15-imino-3,5-metheno-7,10:20,17-dinitrilo-1,2-dioxeto[3',4':3,4]-cyclo-pent[1,2-b]-azacyclononadecine-19-propanoic acid	bidenphytin B	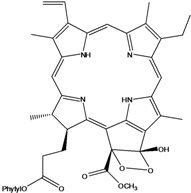	LF	Taiwan	[90]
**188**	(2*E*,7*R*,11*R*)-3,7,11,15-tetramethyl-2-hexadecen-1-yl ester-(3*R*,4*S*,21*R*)-14-ethyl-21-hydroxy-21-(methoxycarbonyl)-4,8,9,13,18-penta-methyl-20-oxo-3-phorbinepropanoic acid	(13^2^*R*)-13^2^-hydroxy-pheophytin a	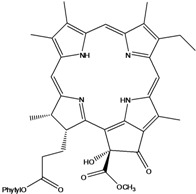	LF	Taiwan	[90]
**189**	(2*E*,7*R*,11*R*)-3,7,11,15-tetramethyl-2-hexadecen-1-yl ester-(3*R*,4*S*,21*S*)-14-ethyl-21-hydroxy-21-(methoxycarbonyl)-4,8,9,13,18-pentamethyl-20-oxo-3-phorbinepropanoic acid	(13^2^*S*)-13^2^-hydroxy-pheophytin a	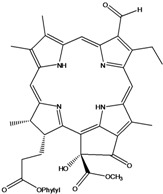	LF	Taiwan	[90]
**190**	(2*E*,7*R*,11*R*)-3,7,11,15-tetramethyl-2-hexa-decen-1-yl ester-(3*R*,4*S*,21*R*)-14-ethyl-13-formyl-21-hydroxy-21-(methoxycarbonyl)-4,8,9,18-tetramethyl-20-oxo-3-phorbine-propanoic acid,	(13^2^*R*)-13^2^-hydroxy-pheophytin b	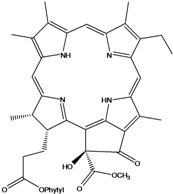	LF	Taiwan	[90]
**191**	(2*E*,7*R*,11*R*)-3,7,11,15-tetramethyl-2-hexadecen-1-yl ester-(3*R*,4*S*,21*S*)-14-ethyl-13-formyl-21-hydroxy-21-(methoxycarbonyl)-4,8,9,18-tetramethyl-20-oxo-3-phorbine-propanoic acid	(13^2 ^*S*)-13^2^-hydroxy-pheophytin b	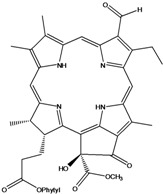	LF	Taiwan	[90]
**192**	(2*E*,7*R*,11*R*)-3,7,11,15-tetramethyl-2-hexa-decen-1-yl ester-(3*S*,4*S*,21*R*)-9-ethenyl-14-ethyl-21-(methoxy-carbonyl)-4,8,13,18-tetramethyl-20-oxo-3-phorbinepropanoic acid	pheophytin a	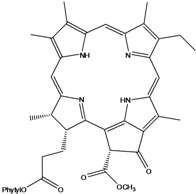	LF	Taiwan	[90]
***Nitrogen and Sulphur-containing Natural Products***						
**193**	3,7-dihydro-1,3,7-trimethyl-1H-purine-2,6-dione	caffeine	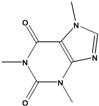	AP	Uganda	[110]
**194**	thymidine		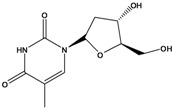	NF	China	[99]
**195**	1-(2-thienyl)-ethanone	2-acetyl-thiophene	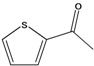	RT	Germany	[122]
***Carbohydrates/ disaccharides***						
**196**		heptanyl 2-*O-β*-xylofuranosyl-(1→6)-*β*-glucopyranoside	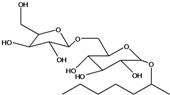	EP	Taiwan	[79]
***Miscellaneous***						
**197**	2-[(3*R*,7*R*,11*R*)-3-hydroxy-3,7,11,15-tetramethylhexadecyl]-3,5,6-trimethyl-2,5-cyclohexadiene-1,4-dione	α-tocopheryl quinone	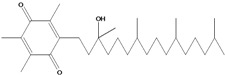	EP	Taiwan	[85]
**198**		7-*O*-(4”,6”-diacetyl)-*β*-*D*-glucopyranoside	NF	LF	China	[59]

AP, Aerial part; LF, Leaf; ST, Steam; EP, Entire plant; FL, Flowers; RT, Root; SD, Seed; LTC, Leaves of tissue culture; PNS, Part not specified; NF, Not found.

### Acetylene compounds

The acetylenes are one class of aliphatic hydrocarbons that has a taxonomically interesting distribution pattern in higher plant families; they occur regularly in only five families, namely the Campanulaceae, Asteraceae, Araliaceae, Pittosporaceae and Umbelliferae [95]. Within the Asteraceae family, these compounds are widely distributed in the Heliantheae tribe [2,4]. The genus *Bidens* is known to produce compounds of this class [5]. They occur in all parts of the plant, often accumulating in roots [96].

To date 34 acetylenes (compounds **35–68**) were isolated from *B. pilosa* (Table 1). The C_13_-polyacetylenes are the most abundant in the species and among them, ene-tetryn-ene **36** and its alcohol, acetyl and aldehyde oxygenated derivatives **40–42**, C_13_-phenylacetylenes **59–66** and C_13_-acetylenes with an ene-triyn-diene chromophore **39 **are typical constituents within the genus *Bidens* [2,4,96,97].

The principal representative of the C_13_-polyacetylenes is 1-phenylhepta-1,3,5-triyne (**64**). This C_13_-phenylacetylene is abundant in *B. pilosa* and is present in leaves, stems and roots of the species [5,73,96,97]. The compound is biologically active and several studies have reported that it strongly absorbs long-wave UV radiation, and the activity is altered upon exposure to light (photo activation) [98]. 

The occurrence of C_17_-acetylenes is rare in the genus, being limited to the Hawaiian species of *Bidens* [4], while one compound (**35**) was related to *B. pilosa* grown in China [2,99]. Also, three C_14_-acetylenes **39,44,46**, with one (**46**) being common in species of genus *Coreopsis*, and another (**44**), a new compound, were reported first in *B. pilosa* [4,51,100].

Another group of polyacetylenes isolated from *B. pilosa* are the polyacetylene glucosides (PAGs), which are glycosides of polyacetylenes in which a sugar moiety (glycose or rhamnose) is joined to a polyacetylene through an -*O*- glucosidic linkage. Of even more restricted distribution, these have been reported for only two families, Asteraceae and Campanulaceae. So far 22 PAGs are known, however most of them have been isolated from *Bidens* species [101].

Studies report the isolation of nine PAGs (**50–58**) from different parts from *B. pilosa*. Four compounds (**50, 53–55**) have the common C_13_-acetylene linkage to glycoside portion in the C_2_ position [49,54,61,102], however the glycoside derivates of C_14_-acetylene have the linkage to the glycoside portion in the terminal portion (**52**) and C3 (**51**) [53,54]. Other unusual three PAGs have also been reported for *B. pilosa*. Two C_16_-acetylenes (**56,57**) and one C_17_-acetylene (**58**) having an ester in the terminal portion linkage to a carboxylic acid [70].

Phenylthiophenes, classified as C_13_-acetylene and related compounds [4], are related to only occur in *Coreopsis* and in Hawaiian *Bidens* [4,103], however a phenylthiophene **67** and its glycosylate **68** were reported for *B. pilosa* growing in China [100].

### Flavonoids

Flavonoids are the class of compound of higher occurrence in the species and are described as chemotaxonomic markers at lower hierarchical levels of the Asteraceae [104]. According to the *Bidens* genus, the flavonoid profile of *B. pilosa* is a complex one that includes aurones, chalcones, flavanones, flavones and flavonols with a wide variety of *O*-methylation patterns and glycosylations [105], totaling 58 different compounds isolated to date (Table 1). 

Anthochlors (aurones and chalcones) are found in a number of plant families, including the Asteraceae. However research indicates that, despite some variations, anthochlors are good markers for the taxonomic subtribe Coreopsidinae (Heliantheae tribe), thus representing the only case in the family Asteraceae in which a certain type of flavonoid is taxonomically diagnostic at the sub tribal level [106]. 

Species of *Bidens* typically contain the chalcones butein (3,4,3’,4’-tetrahydroxychalcone, **112**), okanin (3,4,2’,3’,4’-pentahydroxychalcone, **113**) and their 4’-glycosides [3]. Of the aurones, maritimetin (6,7,3',4'-tetrahydroxyaurone, **102**) and sulfuretin (6,3',4'-tetrahydroxyaurone, **101**) and their glycosides are commonly found in the genus [107]. These compounds have been reported for *B. pilosa* [108]. 

In *B. pilosa,* the glycosides aurones are frequent in position 6 (**103–110**) and rare in 7 **(104**) while the glycosides derived from chalcones (**111,114–128**)are in the positions 3’ and 4’. Two chalcone glycosides, one in position 2’ (**111**) and other in 4 (**128**) were also found to the specie [59,102]. Most of these compounds are acylated with *p-*coumaric and/or acetic acid on the sugar moiety and are relatively non-polar; however more polar aurones (**103,104) **and chalcones (**111,114,115,124,128**), mono- and diglucosides were isolated from aerial parts [109]. Two B-ring methylated chalcones (**126–127**) [80,100] were also found in the species, but this kind of derivatives is rarely reported in the *Bidens* genus [3].

Flavones and flavonols identified from members of *Bidens* are for the most part commonly encountered compounds, *i.e*., glycosides of apigenin, luteolin, kaempferol and quercetin [105]. *B. pilosa* maintains that standard, however some flavonols present methoxy substitutent groups at their positions 3, 6, 7, 3’ and/or 4’, as in jacein (**149**), centaureidin (**139**) and its glycoside centaurein (**140**) [74,79]. Among the flavones 5-*O-*methylhoslundin (**135**) was reported, a compound previously isolated only from *Hoslundia opposite* (Lamiaceae) [110]. This unusual compound presents methoxy substituted groups in C5 and C7 and a pyranone derivative at C6. 

### Other compound classes

Several other compound classes have been isolated from different parts of *B. pilosa* and are listed in Table 1. Among these, aliphatic hydrocarbon derivatives and simple aromatic hydrocarbons have been reported, although these constituents are rather ubiquitous in plants.Long chain saturated unbranched hydrocarbons between C_21_ and C_33_ (**1–13**) have been isolated of *B. pilosa* [44,91]. Of the saturated unbranched alcohols, the compound 2-butoxyethanol (**14**) is the only ether-ethanol, while for the unbranched aliphatic carboxylic acid and ester group, three compounds have ether-ester functions (**32–34**). The simple aromatic hydrocarbons and simple phenylpropanoid compounds form two small groups of natural products in *B. pilosa*. In the first, vanillic (**80**), salicylic (**78**) and protocatechuic (**79**) acids and their derivatives are predominant [87], while the phenylpropanoids are represented by coumaric (**82**), ferulic (**84**) and caffeic (**85**) acid. In this group, one new disubstituted acetylacetone (**86**) was described for *B. pilosa* growing in India [90].

Also in the phenylpropanoids group, caffeoyl ester derivatives **87–97** are fairly reported for the specie, and some esters formed by the combination of two caffeic acids to one quinic acid (**93–97**) [79,83] or one caffeic acid to one erythronic acid (**88–92**) [88]. The only coumarin (**100**) described for *B. pilosa* is usually found in other species of the family [86].

Of the mevalonate pathway, several sesquiterpenes (**160–167**), sterols (**171–177**) and triterpenes (**178–183**) have been isolated of leaves from *B. pilosa* [44,51,86]. The sesquiterpenes reported were characterized by GC-MS [46]. These are divided into mono- and bicyclic, commonly found in leaf extracts from Asteraceae. In the diterpenes, acyclic phytane diterpenoids have been reported; among them phytyl heptanoate (**170**) is an unusual compound that has an aliphatic chain of seven carbon atoms linked to the terminal acid portion [84].

The most abundant sterols from *B. pilosa* are stigmasterol (**177**) and sitosterol (**173**), which are ubiquitous compounds of plant cell membranes [111]. Stigmasterol derivates (**175,176**), sitosterol glucoside (**174**) [85,91] and phytosterin B (**172**), a phytosterin first isolated in *B. pilosa* [112] has also been reported. Among the triterpenes, only squalene (**183**) is an acyclic one. The friedelanes **181,182** and lupeol derivatives **178, 179** are the more common triterpenes reported for *B. pilosa* [44,86]. Among the tetraterpenes *β*-carotene (**184**) is reported to be present in high concentration in young leaves of *B. pilosa* [113].

Chlorin (=2,3-dihydroporphyrin) and its derivatives – including chlorophyll, pheophytin, chlorophyllin, pheophobide, and many other closely related analogues – are found in most higher plants, algae, and even bacteria [114]. For *B. pilosa* two new pheophytins (**186,187**), with peroxide functionalities in ring *E* were reported, besides another six pheophytins (**185,188–192**), already known [114].

Only two representatives of the class of nitrogen-containing natural products, one being the nucleoside thymidine (**194**) are reported [122]. One thyophene (**195**) was reported from *B. pilosa* [99]. One disaccharide (**196**) was isolated from an entire *B. pilosa.* Also, two miscellaneous representatives were reported, a quinone linked to an aliphatic chain (**197**) [85] and one compound of unidentified structure (**198**) [59].

The content of essential oil from flowers, leaves and stems of *B. pilosa* has been analyzed by GC-MS in China, Japan, USA, Cameroon, Nigeria and Iran [66,115,116,117,118,119,120,136]. In this review, the series of components identified as being commonly found in plants containing essential oils and present mostly in very small quantities are not listed. It is then just a brief comment about the main and unusual constituents. In the species a series of mono- and sesquiterpenes have been detected [66,116,117,118,119]. The major constituents are the sesquiterpenes germacrene-D and *β*-caryophyllene. Polyacetylenes (**36,59,60,64**), including 1-phenylhepta-1,3,5-tryin (**64**) have been identified in root oil and aerial parts [117,119]. A chromone, known as precocene I, isolated from oil of the leaves from *B. pilosa* also was reported [116].
